# Topological Superfluid and Majorana Zero Modes in Synthetic Dimension

**DOI:** 10.1038/srep15927

**Published:** 2015-10-30

**Authors:** Zhongbo Yan, Shaolong Wan, Zhong Wang

**Affiliations:** 1 Institute for Advanced Study, Tsinghua University, Beijing, 100084, China; 2 Institute for Theoretical Physics and Department of Modern Physics University of Science and Technology of China, Hefei, 230026, P.R. China; 3 Collaborative Innovation Center of Quantum Matter, Beijing 100871, China

## Abstract

Recently it has been shown that multicomponent spin-orbit-coupled fermions in one-dimensional optical lattices can be viewed as spinless fermions moving in two-dimensional synthetic lattices with synthetic magnetic flux. The quantum Hall edge states in these systems have been observed in recent experiments. In this paper we study the effect of an attractive Hubbard interaction. Since the Hubbard interaction is long-range in the synthetic dimension, it is able to efficiently induce Cooper pairing between the counterpropagating chiral edge states. The topological class of the resultant one-dimensional superfluid is determined by the parity (even/odd) of the Chern number in the two-dimensional synthetic lattice. We also show the presence of a chiral symmetry in our model, which implies Z classification and the robustness of multiple zero modes when this symmetry is unbroken.

Topological superconductors and topological superfluids hosting Majorana zero modes^1^,^2^,^3^,^4^,^5^,^6^,^7^ have been among the central themes of both condensed matter and cold atom physics recently. (“Topological superconductor” refers to charged particles, while “topological superfluid” refers to neutral particles, otherwise their physics is essentially the same. The result of our paper is equally applicable to topological superconductors and topological superfluids.) Apart from being novel phases of matter, they have potential applications in quantum computation^8^,^9^. It is therefore highly desirable to search for various routes towards realization of topological superconductivity/superfluidity and Majorana zero modes. There have been several proposals to realize them in either condensed matter^10^,^11^,^12^,^13^,^14^,^15^,^16^,^17^,^18^,^19^ or cold atom systems^20^,^21^,^22^,^23^. The latter systems have the advantage of high controllability. Experimental study on topological superconductors and Majorana zero modes is also extremely active^24^,^25^,^26^,^27^,^28^.

In this paper we study the Cooper pairing between chiral edge modes of quantum Hall strips as a possible route toward one-dimensional (1D) topological superconductors and topological superfluids. This is stimulated by the idea of “synthetic dimension”^29^,^30^ emerging from optical lattice with atoms with large spin^31^,^32^,^33^,^34^,^35^,^36^,^37^,^38^,^39^,^40^,^41^,^42^. In this visualization, internal degrees of freedom (“spin”) form an additional spatial dimension. This picture is especially convenient when the internal states are coupled sequentially, as can be readily done by two Raman beams^29^. Synthetic magnetic flux naturally exists inside the two-dimensional (one physical dimension plus one synthetic dimension) lattice, and the quantum Hall states (Chern insulator) can be simulated. The chiral edge modes have been observed in recent experiments^43^,^44^. The two counterpropagating chiral modes are separated in the synthetic dimension, therefore they are immune to backscattering if the scatters are short-range.

The interaction effect in this system is an important issue^29^,^45^,^46^,^47^, both experimentally and theoretically. With the motivation of realizing topological superfluidity, in this paper we study the effect of an attractive Hubbard interaction, which is long-range in the synthetic dimension, though short-range in the physical dimension. As a consequence, the two counterpropagating chiral edge modes can be Cooper-paired efficiently. We find that topological superfluidity naturally arises. We also study the existence of topological Majorana zero modes. Interestingly, multiple zero modes are stable if a chiral symmetry of our model is unbroken.

## Results

### The model and main picture

**Figure 1:**
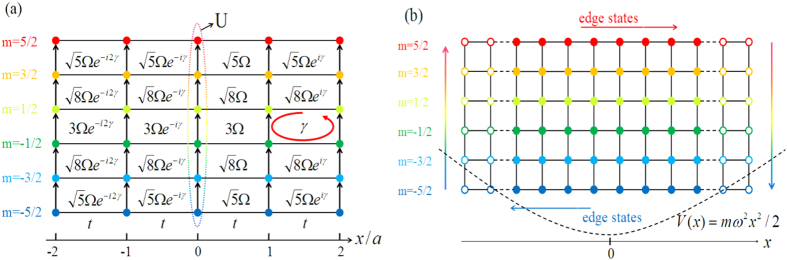
Sketch of the system. The spin states are coupled sequentially by Raman-induced hopping, which generates a synthetic dimension, in addition to the physical dimension *x*.

The system in consideration is illustrated in Fig. 1. The one-dimensional optical lattice extends in the *x* direction, and the Raman-induced hopping couples 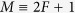 spin states at each site *x* in a sequential manner (*F* = 5/2 is shown in Fig. 1). Therefore, the system acquires a “synthetic dimension”^29^. The Hamiltonian is 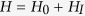, in which the free part is


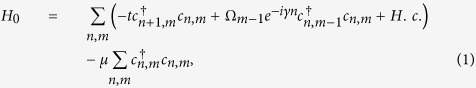


where 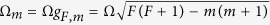
29, with Ω depending on the strength of Raman transitions. Generally, the value of Ω_*m*_ can be controlled by tuning Raman beams. Our results will be insensitive to details of Ω_*m*_. The above Hamiltonian is readily realizable in experiment^43^,^44^. The presence of 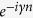 indicates that there is a flux *γ* in each plaquette, which is responsible for the emergence of the chiral edge states in this model(see Fig. (1)). After a gauge transformation 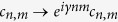, *H*_0_ becomes


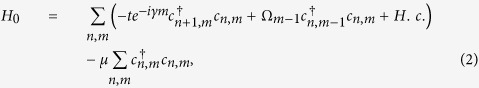


in which the hopping gains a spin-dependent phase factor.

In this paper we take the simple yet realistic SU(*M*)-invariant Hubbard interaction:


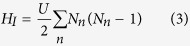


where 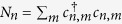 and 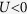. This interaction is apparently long-range in the synthetic dimension, thus it is quite capable of pairing the counterpropagating modes at the opposite edges (near 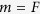 and 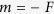, respectively) in the synthetic dimension.

**Figure 2:**
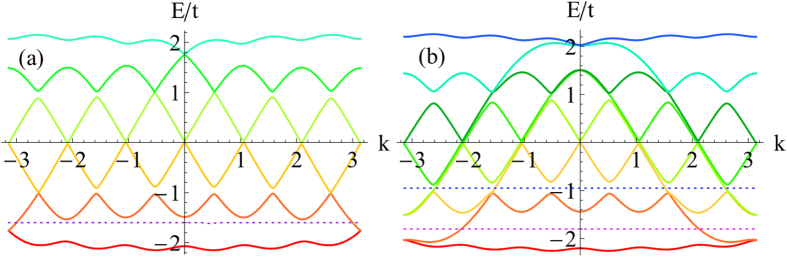
The spectrum of free Hamiltonian *H*_0_ for (a) *F* = 5/2, *t* = 1, Ω = 0.1, *γ* = *π*/3. The dotted line is located at 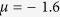; and (**b**) 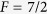, *t* = 1, Ω = 0.1, 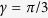. The purple dotted line is located at 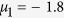, and the blue dotted line is located at 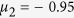.

Before proceeding to a quantitative study of *H*_*I*_, we would like to discuss the physical picture of possible topological superfluidity in this model. Since the hopping lacks translational symmetry along the synthetic dimension, the bulk Chern number as an integral of Berry curvature in the two-dimensional Brillouin zone cannot be defined, however, its manifestation as the number of chiral edge modes is well-defined. Suppose that the “bulk Chern number” *C* = 1, i.e. there is a single pair of chiral edge modes in the bulk gap [see Fig. 2, in which the purple dotted lines intersect with the chiral modes]. If there is a small Cooper pairing between these two edge modes, the system is a one-dimensional topological superfluid. This can be inferred using Kitaev’s *Z*_2_ topological invariant^2^, which essentially counts the parity (even/odd) of the number of Fermi points within 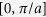 in the absence of pairing. In the case 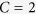, i.e. there are two pairs of chiral edge modes, as illustrated by the curves intersecting with the blue dotted line in Fig. (2b), the superfluid resulting from pairing the edge states is topologically trivial. Provided that the pairing is small, the superfluid (or superconductor) is nontrivial(trivial) when the bulk Chern number is odd(even), namely,


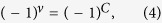


where 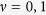 (mod 2) is the Z_2_ topological number of 1D superconductor or superfluid^2^. In the rest part of this paper we shall present a quantitative study of the picture outlined above.

### Cooper pairing in self-consistent mean-field

At the mean-field level the Hubbard interaction can be decomposed as


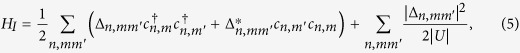


where we have defined 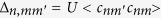, which satisfies 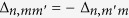 because of Fermi statistics. The mean field BdG Hamiltonian becomes


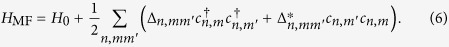


Since the basic physics is the pairing of chiral edge modes with opposite momenta, it is natural to consider Cooper pairing with zero total momentum, namely that 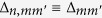 is independent of *n*. We determine these 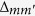 self-consistently. Whenever there are several sets of self-consistent solutions of 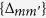, we compare their mean-field energies and pick up the ground state. This calculation can be carried out for all possible values of *F*. Hereafter we take *F* = 7/2 as an example.

**Figure 3:**
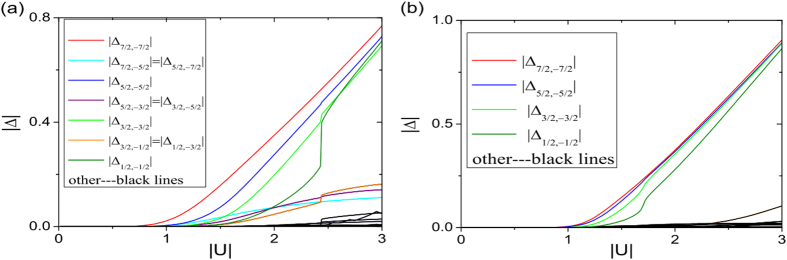
The self-consistent solutions of Cooper pairing 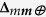 as functions of *U* for (a) *μ*_1_ **= −1.8; (b)** ***μ***_**2**_** = −0.95.** The parameters are *t* = 1, Ω = 0.1, and *γ* = *π*/3, which are the same as used in Fig. (2b).

The pairings as functions of Hubbard *U* are shown in Fig. (3) for two values of chemical potential *μ*. In Fig. (3a) we take 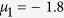, which corresponds to bulk Chern number *C* = 1 [see the purple dotted line in Fig. (2b)]. For small 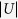, as 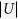 increases, 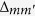 grows exponentially. For *U* not too large, 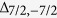 dominates other pairings, which is consistent with the picture of pairing between chiral edge modes. At a critical 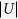 slightly below 2.5, the system undergoes a first-order transition to a phase in which 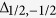, which should be regarded as a “bulk pairing”, becomes comparable to the edge pairing. Thus the edge-pairing picture becomes inaccurate at large 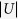. In Fig. (3b) a different chemical potential 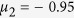 is taken, and the edge-state pairings again dominate at small 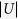.

**Figure 4:**
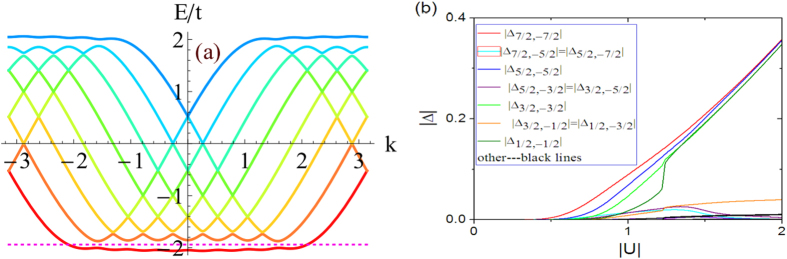
(**a**) The spectrum without interaction for *t* = 1, 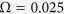, and 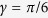. The dashed line marks 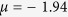. (**b**) The Cooper pairings as functions of *U*.

To illustrate the robustness of the present picture, we study a set of quite different parameters, shown in Fig. (4). The behavior of pairings is qualitatively the same as that for the previous parameters.

A few remarks are in order. First, although we have taken the strength of hopping along the synthetic dimension as 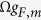, we have also checked that the result is qualitatively the same when it is *m*-independent. Second, when the 2D bulk is metallic, topological superfluidity can still emerge, though there is no clear criteria using Chern number. We shall not focus on details about this.

### Majorana zero modes

The hallmark of 1D topological superconductor or superfluid is the emergence of topological zero modes localized near the two ends of an open chain.

**Figure 5:**
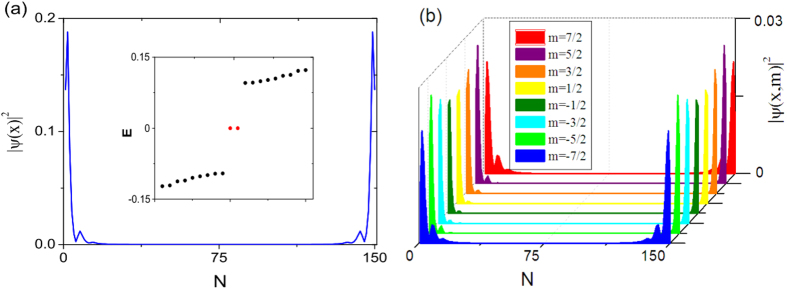
(**a**) The wavefunction of a zero mode in an open chain with length *N* = 150. The parameters are 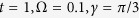 and 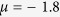. The Cooper pairings are taken as 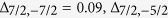

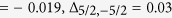
, 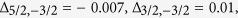

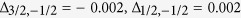
, which are the mean-field pairing obtained at 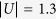 ( see also Fig. (3a)). Other 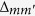
’s are much smaller and thus neglected. The two zero modes have the same profile of 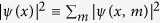, thus only one is shown here. The inset shows a few energies near *E* = 0. (**b**) The zero mode solution with *m* resolution.

We have solved the BdG mean-field Hamiltonian Eq.(6) for the wavefunction 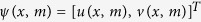, in which 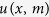 and 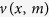 denote the particle and hole component respectively. Below we present our solutions in a chain with sharp boundary, for the parameters *t* = 1, 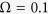, 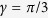, at both 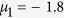 and 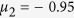. The Cooper pairings are taken to be the mean-field values at 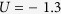, which we obtained in the previous section. The case 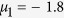 is shown in Fig. 5. There is one zero mode localized at each end of the open chain, and a tiny finite-size coupling mixes them slightly, though the energy splitting due to finite-size effect is too small to be discernable. The existence of a single Majorana zero modes at each end of an open chain is consistent with Eq.(4), the bulk Chern number being *C* = 1 (odd number) at 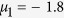. The superfluid is topologically nontrivial in this case.

**Figure 6:**
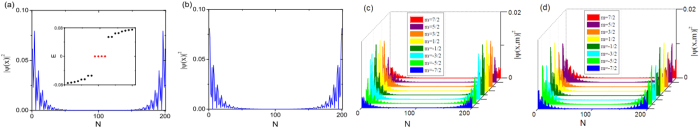
The wavefunction of zero modes in an open chain with length *N* = 200. The parameters are 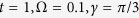 and 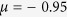, which is the same as Fig. 5 except for the chemical potential. The pairings are 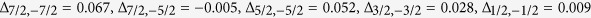 (the mean-field pairing at 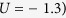, and other pairing terms are much smaller and thus neglected. The spatial profile of 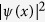 of two of the four zero modes are shown in (**a**,**b**), while the profile with *m* resolution is shown in (**c**,**d**). The other two zero modes with the same profiles are not shown repeatedly. The inset of (**a**) shows several energies close to *E* = 0.

As a comparison, we also present the zero mode solutions at 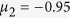, for which the free Hamiltonian have two pairs of chiral edge modes (*C* = 2), indicating that the superfluid phase at small 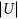 should be *Z*_2_ topologically trivial (see Eq.(4)). In the numerical calculation with open boundary condition, we find two Majorana zero modes at each end (see Fig. 6), which means that the superfluid is Z_2_ trivial. Therefore we see again that (−1)^*C*^ determines the *Z*_2_ topological classification of the 1D superfluid in synthetic dimension.

One may wonder why there is no hybridization between the two zero modes, which may open a gap for them. We shall explain the reason as follows. In fact, the BdG Hamiltonian has a time-reversal symmetry and a particle-hole symmetry, which can be combined into a chiral symmetry^48^. If the Cooper pairing 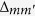 are real, then we can check that the Hamiltonian satisfies


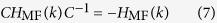


in which


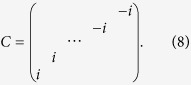


This matrix is written in the BdG basis of 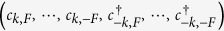
^T^. Due to these symmetries, the BdG Hamiltonian can be classified as BDI^48^,^49^, whose classification in 1D is Z. The *Z*_2_ topologically trivial phase is nontrivial according to the *Z* classification of BDI class, which is the reason why zero modes appear at the edge of *Z*_2_ trivial states. We have checked that, if we break the symmetries, e.g. by giving a phase factor to 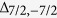 (with other 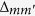
s unchanged), then these zero modes will be shifted to nonzero energies.

According to Fidkowski and Kitaev’s work^50^, in the presence of interaction, the classification of BDI-class topological superconductors in 1D is Z_8_ instead of Z. Because of the flexible tunability to topological superconductors with large topological number (say 8) in our system, the Z_8_ classification can be tested experimentally. If we tune the bulk Chern number of our system to 8, the eight nominally-zero-modes will be shifted away from zero energy due to the (beyond-mean-field) interaction effects of the Hubbard term. If observed, this will be an experimental test of Fidkowski and Kitaev’s Z_8_ classification.

**Figure 7:**
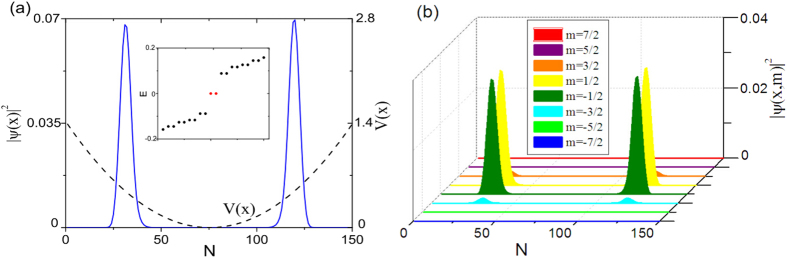
Majorana zero modes in the harmonic trap. The parameters are *t* = 1, Ω = 0.1, and 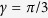, which are the same as used in Fig. (2b). The harmonic trap is 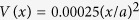, where *x* = 0 is the center of the chain with size *N* = 150. The two zero modes have the same profile of 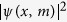, thus we only show one. The inset of (**a**) shows several energies close to *E* = 0.

To make a closer connection to experiment, we also study the existence of Majorana zero modes in a system with soft boundary created by a harmonic trap *V*(*x*). In the presence of *V*(*x*), the chemical potential becomes 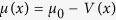. We take 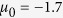 and 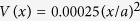, such that the center of the system is topologically nontrivial. Since 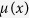 is not constant, 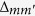 should also be *x*-dependent. To incorporate this effect, we numerically calculate the functions 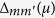 at 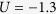, which is then used to produce the mean-field BdG Hamiltonian in harmonic trap. In the solution to this BdG Hamiltonian, the zero modes can be clearly seen, as shown in Fig. (7), though the quantitative details are different from the case of hard boundary.

## Conclusions and Discussions

We have studied the pairing between counterpropagating chiral edge modes in the quantum Hall strip in synthetic dimension. This picture has several merits. Creation of magnetic flux in the synthetic dimension by Raman beans is easier than in physical dimensions. The spatial separation of left and right moving chiral edge states in the synthetic dimension effectively prevents the backscattering between them, which implies their robustness. Meanwhile, the Hubbard interaction is not suppressed by this spatial separation: It is infinite-range in the synthetic dimension, therefore, it can pair the two edge modes quite efficiently. As we have shown, the resultant states are topological superfluid carrying Majorana zero modes. If the chiral symmetry of our model is unbroken, the classification is Z and multiple zero modes are stable; on the other hand, if this symmetry is broken, the classification is Z_2_.

Finally, we remark that quantum fluctuations of the phase factor of pairing in 1D is generally strong. One can put the 1D system in proximity to a 3D supefluid to suppress these fluctuations^20^. Moreover, it has been shown^51^,^52^ that long-range superconducting order is not a necessary condition for the existence of Majorana zero modes. The zero modes persist even when the long-range superconducting order is replaced by algebraic order (i.e. the correlations of pairings decay by power-law). In our system this conclusion applies.

## Methods

### Mean-field calculations

The mean-field calculation is carried out by the standard procedure of decomposing the Hubbard interaction as fermion bilinear terms, leading to Eq.(6). The Cooper pairing is calculated from Eq.(6) in a self-consistent manner. All self-consistent solutions for the Cooper pairing are obtained. In the case that there are more than one self-consistent solutions, the one with lowest mean-field energy is selected.

## Additional Information

**How to cite this article**: Yan, Z. *et al.* Topological Superfluid and Majorana Zero Modes in Synthetic Dimension. *Sci. Rep.* **5**, 15927; doi: 10.1038/srep15927 (2015).
